# High CRP and white blood cell counts are not reliable indicators of early-onset neonatal infection in full-term infants

**DOI:** 10.3389/fped.2025.1707339

**Published:** 2026-01-15

**Authors:** Liang Liu, Xueou Liu, Lulu Zhang, Junling Ma, Fangrui Ding

**Affiliations:** 1 Department of Neonatology, Tianjin Central Hospital of Gynecology Obstetrics, Tianjin, China; 2 Tianjin Key Laboratory of Human Development and Reproductive Regulation, Tianjin, China; 3 Department of Neonatology, Nankai University Maternity Hospital, Tianjin, China; 4 Tianjin Institute of Gynecology Obstetrics, Tianjin Central Hospital of Gynecology Obstetrics, Tianjin, China

**Keywords:** antibiotic stewardship, biomarkers, infectious disease, neonate, sepsis

## Abstract

**Background:**

Diagnosing neonatal early-onset sepsis (EOS) is challenging, making it difficult to determine infection indicator characteristics and cutoff values in full-term infants. This study retrospectively analyzed full-term infants with high-risk factors for neonatal EOS but tested negative for EOS, aiming to identify infection indicator characteristics and their association with perinatal factors without antibiotic intervention.

**Methods:**

Full-term infants at high risk for EOS who were admitted to rooming-in from 1 July 2023 to 29 February 2024 were included in the study. Blood routine examinations and C-reactive protein (CRP) levels were dynamically monitored after birth. All demographic data and medical records were collected from the electronic medical records system.

**Results:**

Among 103 neonates, only 2 had normal infection indicators. Within 24–48 h after birth, an additional 28 displayed normal infection indicators. Although most of the infants exhibited normal WBC within 24–48 h, 33 patients still had higher neutrophil percentages, and 62 had higher CRP levels. Moreover, several high-risk perinatal factors for abnormal infection indicators have been identified.

**Conclusions:**

This study demonstrates that infection indicators frequently showed abnormalities in full-term infants at high risk for EOS who tested negative. Few infants had normal infection indicators within 24 h, and although WBC levels normalized by 24–48 h, CRP levels remained elevated. Specific perinatal factors were also associated with abnormal infection markers. These findings reinforce the need for cautious interpretation of isolated biomarker elevations and support antimicrobial stewardship by highlighting the high frequency of abnormal indicators in uninfected infants, thereby reducing unnecessary antibiotic exposure.

## 1 Introduction

Neonatal early-onset sepsis (EOS) is one of the leading causes of neonatal mortality (1, 2). Prophylactic antibiotic use in neonates with high-risk factors can effectively prevent neonatal sepsis and reduce both mortality and morbidity (3–6). However, antibiotic overuse also warrants attention, as it can disrupt the intestinal microbiome, contribute to antibiotic resistance, and lead to future metabolic disorders and immune dysregulation-related diseases (7–13). The World Health Organization has already prioritized antibiotic resistance as part of its global action plan (14).

The gold standard for diagnosing EOS is the positive identification of a pathogenic organism from sterile body fluid (4–6). However, current studies have demonstrated that pathogen cultures have low sensitivity, making the diagnosis of neonatal sepsis challenging (15–19). Furthermore, although many laboratories provide preliminary blood culture results within 36–48 h, final pathogen identification and susceptibility testing often require additional time (15–19). If antibiotic administration is based solely on this gold standard, suspected sepsis may rapidly progress to life-threatening outcomes by the time culture results become available. In recent years, molecular diagnostic techniques such as polymerase chain reaction (PCR) have been increasingly utilized to aid in the rapid detection of pathogens, although their integration into routine neonatal sepsis evaluations varies across settings. Consequently, clinical suspicion and infection indicators often guide antibiotic treatment decisions (20–23). Common blood infection indicators include white blood cell (WBC) count, neutrophil percentage, C-reactive protein (CRP), procalcitonin (PCT), and interleukin-6 (IL-6). However, these indicators can also respond to early acute stress events, such as delivery, respiratory distress, and hypoxic stress in neonates, which, in turn, may influence infection indicators (24–28). This makes it difficult to decide whether antibiotics should be used in these cases. To date, there are no definitive inflammatory markers or cutoff values for infection indicators in neonatal EOS that clearly indicate whether antibiotics should be administered (3, 4, 16). Once antibiotics are used, these neonates are hospitalized, separated from their mothers, and face increased healthcare costs.

In fact, research in this field is particularly challenging because infection markers in infants suspected of having, but not actually diagnosed with, EOS may be affected by prior antibiotic use. Therefore, in the present study, we took an alternative approach. We retrospectively collected data on full-term infants with high-risk factors for EOS who were ultimately EOS-negative and did not receive antibiotics. We analyzed their infection indicators and relevant perinatal factors, aiming to offer valuable insights for guiding future antibiotic use in this population.

## 2 Methods

The Ethics Review Committee of Tianjin Central Hospital of Gynecology Obstetrics approved this study (No. 2024KY072). All study protocols adhered to the principles of the Declaration of Helsinki. The requirement for written informed consent was waived due to the retrospective nature of the clinical data collection. This study followed the Strengthening the Reporting of Observational Studies in Epidemiology (STROBE) reporting guidelines.

### 2.1 Study population

This observational study included all full-term live-born newborns at Tianjin Central Hospital of Gynecology Obstetrics (He Ping District) from 1 July 2023 to 29 February 2024. The following newborns were excluded: (1) infants admitted to the NICU after birth, (2) infants admitted to rooming-in but with a gestational age of <37 weeks; (3) infants without a high risk of EOS and without infection indicator monitoring after birth; (4) infants without follow-up information to confirm the presence or absence of EOS; and (5) infants diagnosed with sepsis, suspected sepsis, or clinical sepsis, and who received any antibiotics within the first 7 days postnatally.

### 2.2 Study definitions

Sepsis was defined as a positive culture of bacteria in blood or other body fluids, along with infectious clinical signs and symptoms and abnormal infection indices. Suspected sepsis was defined as the presence of infectious clinical signs and symptoms or maternal chorioamnionitis. Clinical sepsis was defined as abnormal infection indices with infectious clinical signs and symptoms, but with negative bacterial cultures. All newborns were followed up within the first 7 days after birth to exclude sepsis, suspected sepsis, and clinical sepsis. Feeding intolerance was defined as the presence of one or more of the following: gastric residuals >50% of the previous feeding volume, abdominal distension requiring interruption or reduction of feeding, vomiting, or clinically significant regurgitation (29). In accordance with standard clinical microbiology protocols, positive blood cultures would have been reviewed for potential contamination based on organism identity (e.g., coagulase-negative staphylococci), time to positivity, clinical correlation, and serial infection markers. Any culture deemed a contaminant in the absence of clinical or laboratory evidence of infection would have led to exclusion from the sepsis-positive group. In this study, all blood cultures were negative, and thus no such exclusions were necessary.

Newborns admitted to rooming-in with high-risk factors for EOS were monitored for infection indicators and clinical signs and symptoms. The infection indicators included in this study were blood routine examinations and CRP levels. High-risk factors for EOS included meconium staining of amniotic fluid, premature rupture of membranes for >18 h, application of vacuum extractor during delivery, maternal fever within 48 h before delivery, and maternal group B streptococcus (GBS) colonization with adequate intrapartum antibiotic prophylaxis (≥4 h before delivery). An abnormal WBC count was defined as >30 × 10^9^/L or <5 × 10^9^/L. An abnormal neutrophil percentage was defined as >70%, and an abnormal CRP level was defined as >10 mg/L (20, 30–32). Absolute neutrophil counts (ANC) were interpreted using established neonatal reference ranges rather than those applied to older infants or children. Specifically, neonatal neutrophil values were evaluated according to the widely used reference ranges described by Manroe et al. (32). All demographic data including maternal complications, maternal white cell count (WCC) before delivery, and medical records were collected from the electronic medical records system. White blood cell counts were obtained from automated complete blood count analysis and were not manually corrected for nucleated red blood cells, consistent with routine clinical reporting in our institution.

Operational definition of EOS-negative status for study inclusion: For the purposes of this analysis, an infant was considered definitively EOS-negative and eligible for inclusion if they fulfilled all of the following criteria during the 7-day postnatal follow-up period—(1) absence of clinical signs of sepsis; (2) receipt of no antibiotic therapy; and (3) either (a) a negative blood culture result (if obtained) or (b) in the absence of blood culture, documented normalization of initial laboratory abnormalities (WBC, neutrophil%, CRP) concurrent with sustained clinical well-being.

Blood culture protocol: Blood culture was not routinely performed for all asymptomatic high-risk infants in the rooming-in ward, consistent with local antimicrobial stewardship practices. It was obtained based on clinical judgment for a subset of infants (23/103, 22.3%) when specific concerns arose; all cultures were negative.

### 2.3 Statistical analysis

R software 4.2.2 was used to conduct the statistical analysis. To characterize the features of the study neonates, categorical data were presented as frequencies (percentages) and continuous variables as medians (interquartile ranges). As outcome variables, WBC, neutrophil percentage, CRP, and composite infection indicators were evaluated. Univariate analysis was conducted between outcome variables and neonatal baseline characteristics including perinatal factors and general clinical symptoms. Pearson's chi-squared test was used to evaluate differences in distribution between groups, and Fisher's exact test was used when any expected cell frequency was 5 or less. Independent infectious risk factors were determined using a multiple binary logistic regression model. All variables were entered simultaneously into the logistic regression models and treated as categorical variables. Odds ratios and 95% confidence intervals (CI) were calculated. A significance level of *p* < 0.05 was used.

## 3 Results

**Figure 1:**
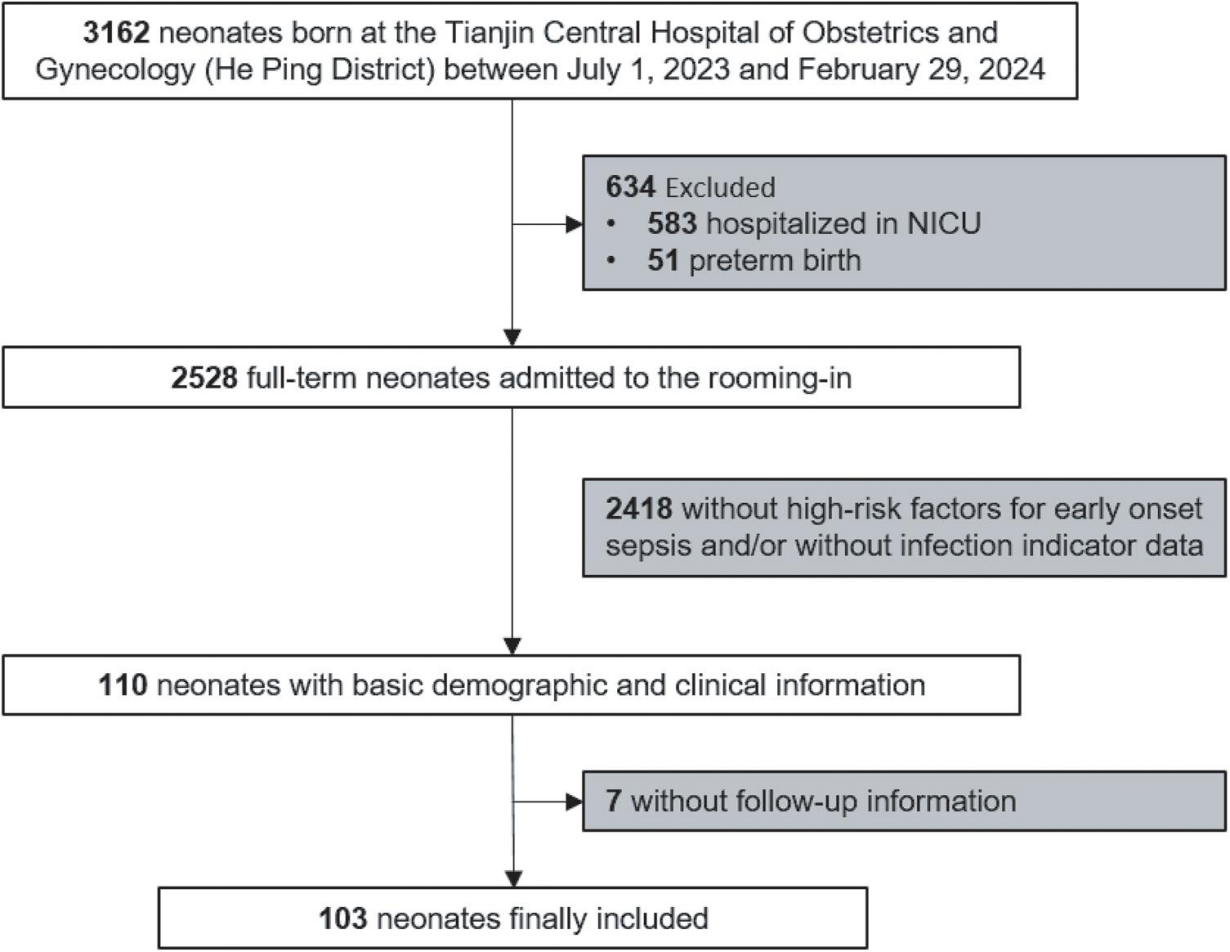
Flowchart for enrolled neonates.

As shown in Figure 1, from July 2023 to February 2024, a total of 3,162 newborns were born at Tianjin Central Hospital of Gynecology Obstetrics (He Ping District), of whom 2,528 were full-term newborns admitted to rooming-in care. Among them, 2,418 infants had no high-risk factors for EOS and/or lacked infection indicator data and were cared for routinely, while 110 infants were identified as having risk factors for EOS. All 110 infants underwent blood routine examinations and CRP testing within 24 h of birth and were closely monitored for clinical symptoms within the first 48 h. Of them, seven infants lacked follow-up information regarding whether they developed infections during the neonatal period. Finally, 103 infants with high risk factors for EOS but ultimately negative for EOS and who did not receive any antibiotics within the first week after birth were included in the present study. Of these 103 infants, blood culture was obtained in 23 (22.3%) based on the doctor’s judgment; all cultures were negative.

As shown in Table 1, the clinical characteristics of the 103 newborns were listed, including not only information about the newborns but also details about their mothers, pregnancy-related factors, and other information closely related to EOS. Information on whether the mother experienced infections during the entire gestation was also included.

**Table 1:** Characteristics of full-term infants with infectious risk factors during the perinatal period.

Characteristics	All
	(*N* = 103)
Neonate sex	
Male	59 (57.3%)
Female	44 (42.7%)
Gestation age, weeks	40.1 (39.6–40.7)
Birth weight, g	3,390 (3,150–3,670)
Maternal age, years	30 (28–33)
Mode of delivery	
Vaginal delivery	72 (69.9%)
Cesarean section	31 (30.1%)
GBS^a^ positive	
Yes	14 (13.6%)
No	89 (86.4%)
Maternal diabetes mellitus	
Yes	20 (19.4%)
No	83 (80.6%)
Abnormality of the placenta^b^	
Yes	10 (9.7%)
No	93 (90.3%)
Abnormality of the umbilical cord^c^	
Yes	38 (36.9%)
No	65 (63.1%)
Infection during pregnancy	
Yes	33 (32.0%)
No	70 (68.0%)
COVID-19 infection during pregnancy	
Yes	18 (17.5%)
No	85 (82.5%)
Meconium staining of amniotic fluid	
Yes	13 (12.6%)
No	90 (87.4%)
Premature rupture of membranes >18 h	
Yes	36 (35.0%)
No	67 (65.0%)
Application of the vacuum extractor during delivery	
Yes	27 (26.2%)
No	76 (73.8%)
Intrapartum fever	
Yes	10 (9.7%)
No	93 (90.3%)
Abnormal FHR monitoring before delivery	
Yes	20 (19.4%)
No	83 (80.6%)
Maternal WBC >10 × 10^9^/L within 48 h before delivery	
Yes	29 (28.2%)
No	74 (71.8%)
Maternal intravenous intrapartum antibiotic	
Yes	56 (54.4%)
No	47 (45.6%)
Neonate feeding intolerance	
Yes	21 (20.4%)
No	82 (79.6%)
Neonatal weight loss >3% on the first day after birth	
Yes	25 (24.3%)
No	78 (75.7%)

All values were shown as *n* (%) or median (interquartile range).

GBS, group B streptococcus; COVID-19, coronavirus disease 2019; FHR, fetal heart rate; WBC, white blood cell.

a GBS positive refers to maternal colonization status (positive rectovaginal group B streptococcal screen during pregnancy).

b Abnormality of the placenta is identified as placental morphology abnormalities, placental size abnormalities, and placental position abnormalities such as placenta abruption, placenta previa, velamentous placenta, and so on.

c Abnormality of the umbilical cord include excessively long umbilical cord, excessively short umbilical cord, umbilical cord entanglement, umbilical cord knotting, umbilical cord twisting, and umbilical cord prolapse.

**Figure 2:**
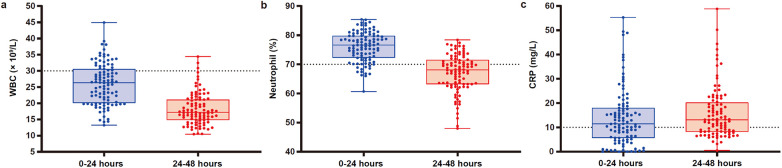
Comparison of neonatal WBC count, neutrophil percentage, and CRP levels between “0–24 h” and “24–48 h” after birth. **(a)** WBC counts (×10^9^/L), **(b)** neutrophil percentage (%), and **(c)** CRP levels (mg/L) were displayed in box plots for neonates measured at two time intervals: 0–24 h (blue) and 24–48 h (red). Each dot represented an individual data point. The central line in each box indicated the median value, the box edges represented the interquartile range, and the whiskers denoted the maximum and minimum values observed within each group. The dotted horizontal lines at 30 × 10^9^/L in panel **a**, 70% in panel **b**, and 10 mg/L in panel **c** indicated clinically relevant thresholds. Observations suggested changes in WBC count, neutrophil percentage, and CRP levels between the two time intervals.

All infants included in the present study underwent blood routine examinations and CRP testing within 24 h and were followed up until their infection indicators normalized. The main infection indicator results, including WBC, neutrophil percentage, and CRP, were shown in Figure 2 and Table 2. Among the 103 neonates, only 2 had completely normal WBC, neutrophil percentage, and CRP results at the initial screening, while the remaining 101 exhibited varying degrees of abnormalities. Specifically, 28 infants had WBC levels above normal (27.7%), 90 had elevated neutrophil percentages (89.1%), and 61 had CRP levels above normal (60.4%).

**Table 2:** Levels of major infection indicators in newborns within 48 h after birth.

Time interval	WBC (×10^9^/L)	Neutrophil percentage (%)	CRP (mg/L)
0–24 h	26.31 (20.25–30.38)	76.60 (72.35–79.80)	11.50 (5.67–17.88)
24–48 h	17.20 (14.89–21.14)	68.10 (63.20–71.45)	13.12 (8.21–19.82)

All values were shown as median (interquartile range).

WBC, white blood cell; CRP, C-reactive protein.

We planned to reexamine blood routine tests and CRP levels in the 101 infants with abnormal results within 24 h after birth. However, 5 of 101 infants were reexamined >48 h after birth due to various reasons. Ultimately, 96 infants were reexamined within 24–48 h after birth. Among these 96 infants, 28 had normal infection indicators. Including the two infants who had normal indicators within the first 24 h, the proportion of infants with normal infection indicators within 48 h after birth was only 29.2%.

In the second examination conducted within 24–48 h after birth, 68 infants still had abnormal infection indicators. Among them, 3 had WBC levels above normal (3.1%), 33 had elevated neutrophil percentages (34.4%), and 62 had CRP levels above normal (64.6%). As shown in Figure 2, WBC and neutrophil percentages exhibited a decreasing trend from 0–24 h to 24–48 h, while CRP levels showed an increasing trend.

**Figure 3:**
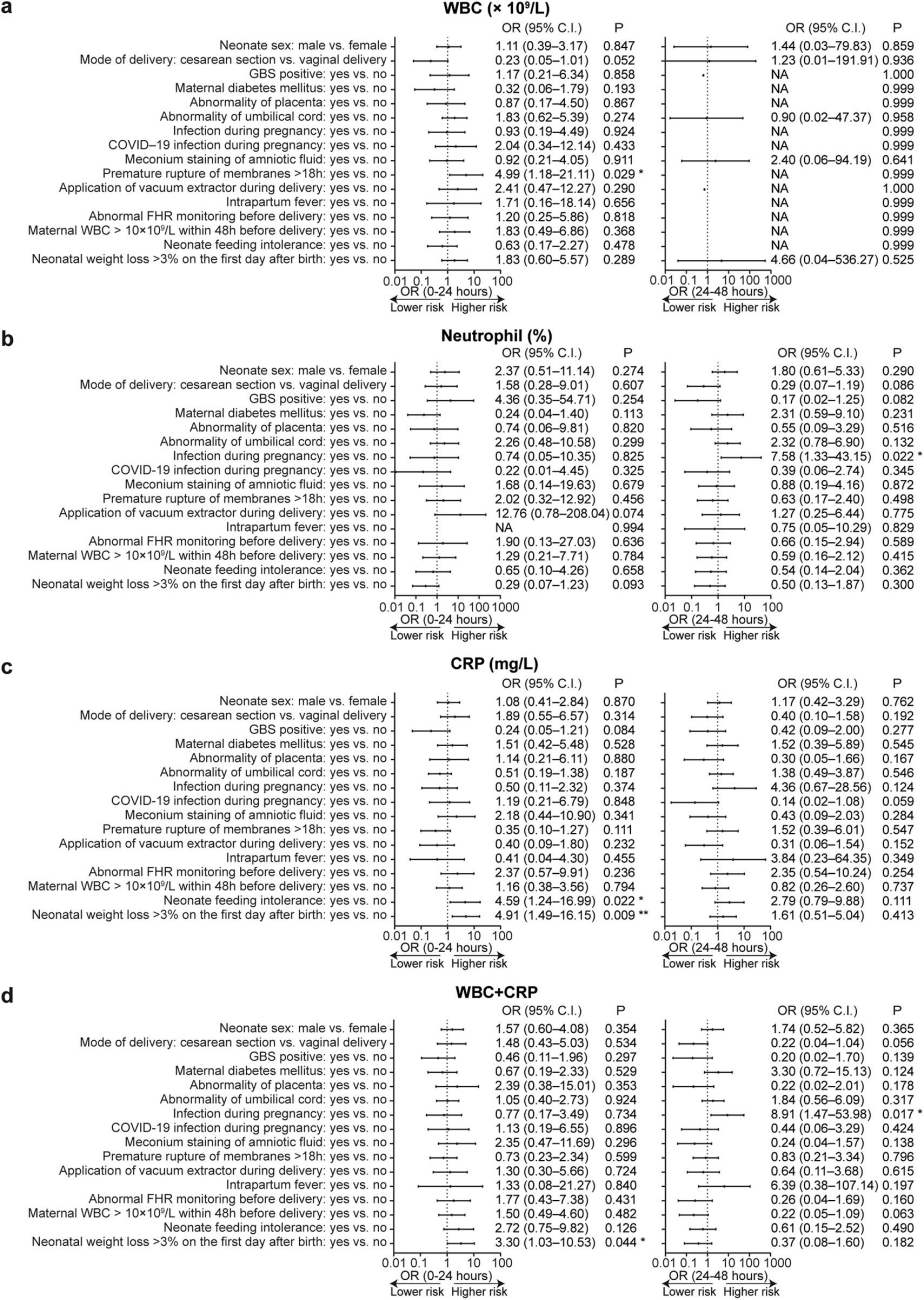
Forest plots showed the odds ratios (OR) for factors associated with neonatal WBC count, neutrophil percentage, and CRP levels at various post-birth time intervals. Panels **a** through **d** presented the ORs with 95% CI for maternal and neonatal factors affecting the following abnormality indicators: **(a)** abnormal WBC count (defined as >30 × 10^9^/L or <5 × 10^9^/L); **(b)** abnormal neutrophil percentage (defined as >70%); **(c)** abnormal CRP levels (defined as >10 mg/L); **(d)** abnormal composite blood index (defined as both WBC > 30 × 10^9^/L and CRP > 10 mg/L). Each panel compared the impact of these factors on neonatal outcomes across specified time intervals (“0–24 h” and “24–48 h”). Multivariate models were fitted using logistic regression. Statistical significance was indicated as follows: *p < 0.05; **p < 0.01. Factors marked as “NA” represented instances where abnormal indicators occurred exclusively within specific subgroups, leading to insufficient sample sizes for accurate estimation of odds ratios.

As previously mentioned, in this cohort, only 30 infants had normal infection indicators within 48 h of birth, with 70.9% showing abnormal indicators. Therefore, we explored the relationship between infection indicators and perinatal factors. As shown in Tables 3 and 4 and Figure 3, within the first 24 h after birth, premature rupture of membranes for >18 h was identified as a high-risk factor for abnormal WBC levels, while neonatal feeding intolerance or weight loss exceeding 3% was a high-risk factor for abnormal CRP levels. According to current studies and recommendations, WBC and CRP are often used together as indicators of infection (33, 34), so we defined composite infection indicator abnormality as having both WBC and CRP outside normal ranges and analyzed the associated outcomes. The study revealed that weight loss exceeding 3% in the first 24 h was a high-risk factor for abnormal composite infection indicators. Additionally, within the 24–48 h after birth, maternal infection during pregnancy was a high-risk factor for abnormal neutrophil percentages and composite infection indicators (WBC + CRP).

**Table 3:** Univariate analysis of the association between perinatal factors, general clinical symptoms, and blood indices within 24 h of birth.

Variable	Abnormal WBC		Abnormal neutrophil percentage		Abnormal CRP		Abnormal composite blood indicators	
	*n*/*N* (%)	*p*-value	*n*/*N* (%)	*p*-value	*n*/*N* (%)	*p*-value	*n*/*N* (%)	*p*-value
Neonate sex		0.491		0.570		0.821		0.640
Male	14/59 (23.7)		53/59 (89.8)		36/59 (61.0)		40/59 (67.8)	
Female	14/44 (31.8)		37/44 (84.1)		25/44 (56.8)		27/44 (61.4)	
Mode of delivery		0.058		>0.99		0.170		0.880
Vaginal delivery	24/72 (33.3)		63/72 (87.5)		39/72 (54.2)		46/72 (63.9)	
Cesarean section	4/31 (12.9)		27/31 (87.1)		22/31 (71.0)		21/31 (67.7)	
GBS positive		0.753		>0.99		0.643		0.554
Yes	3/14 (21.4)		13/14 (92.9)		7/14 (50.0)		8/14 (57.1)	
No	25/89 (28.1)		77/89 (86.5)		54/89 (60.7)		59/89 (66.3)	
Maternal diabetes mellitus		0.100		0.274		0.401		0.790
Yes	2/20 (10.0)		16/20 (80.0)		14/20 (70.0)		12/20 (60.0)	
No	26/83 (31.3)		74/83 (89.2)		47/83 (56.6)		55/83 (66.3)	
Abnormality of the placent^a^		0.454		>0.99		0.737		0.488
Yes	4/10 (40.0)		9/10 (90.0)		5/10 (50.0)		8/10 (80.0)	
No	24/93 (25.8)		81/93 (87.1)		56/93 (60.2)		59/93 (63.4)	
Abnormality of the umbilical cord^b^		0.591		0.763		0.405		>0.99
Yes	12/38 (31.6)		34/38 (89.5)		20/38 (52.6)		25/38 (65.8)	
No	16/65 (24.6)		56/65 (86.2)		41/65 (63.1)		42/65 (64.6)	
Infection during pregnancy		0.230		0.751		0.191		0.988
Yes	12/33 (36.4)		28/33 (84.8)		16/33 (48.5)		22/33 (66.7)	
No	16/70 (22.9)		62/70 (88.6)		45/70 (64.3)		45/70 (64.3)	
COVID-19 infection during pregnancy		0.085		0.235		0.540		>0.99
Yes	8/18 (44.4)		14/18 (77.8)		9/18 (50.0)		12/18 (66.7)	
No	20/85 (23.5)		76/85 (89.4)		52/85 (61.2)		55/85 (64.7)	
Meconium staining of amniotic fluid		0.746		>0.99		>0.99		0.535
Yes	4/13 (30.8)		12/13 (92.3)		8/13 (61.5)		10/13 (76.9)	
No	24/90 (26.7)		78/90 (86.7)		53/90 (58.9)		57/90 (63.3)	
Premature rupture of membranes >18 h		0.426		0.370		0.730		0.691
Yes	12/36 (33.3)		30/36 (83.3)		20/36 (55.6)		22/36 (61.1)	
No	16/67 (23.9)		60/67 (89.6)		41/67 (61.2)		45/67 (67.2)	
Application of the vacuum extractor during delivery		0.277		0.176		0.823		0.363
Yes	10/27 (37.0)		26/27 (96.3)		15/27 (55.6)		20/27 (74.1)	
No	18/76 (23.7)		64/76 (84.2)		46/76 (60.5)		47/76 (61.8)	
Intrapartum fever		>0.99		0.355		0.737		0.488
Yes	3/10 (30.0)		10/10 (100.0)		5/10 (50.0)		8/10 (80.0)	
No	25/93 (26.9)		80/93 (86.0)		56/93 (60.2)		59/93 (63.4)	
Abnormal FHR monitoring before delivery		0.972		0.454		0.740		0.436
Yes	6/20 (30.0)		19/20 (95.0)		13/20 (65.0)		15/20 (75.0)	
No	22/83 (26.5)		71/83 (85.5)		48/83 (57.8)		52/83 (62.7)	
Maternal WBC >10 × 10^9^/L within 48 h before delivery		>0.99		>0.99		0.885		0.770
Yes	8/29 (27.6)		25/29 (86.2)		18/29 (62.1)		20/29 (69.0)	
No	20/74 (27.0)		65/74 (87.8)		43/74 (58.1)		47/74 (63.5)	
Neonate feeding intolerance		0.909		>0.99		0.127		0.145
Yes	5/21 (23.8)		19/21 (90.5)		16/21 (76.2)		17/21 (81.0)	
No	23/82 (28.0)		71/82 (86.6)		45/82 (54.9)		50/82 (61.0)	
Neonatal weight loss >3% on the first day after birth		0.716		0.296		0.028*		0.119
Yes	8/25 (32.0)		20/25 (80.0)		20/25 (80.0)		20/25 (80.0)	
No	20/78 (25.6)		70/78 (89.7)		41/78 (52.6)		47/78 (60.3)	

*p*-values were calculated using Pearson's chi-squared test for comparisons between groups, and Fisher's exact test was used when any expected cell frequency was 5 or less.

GBS, group B streptococcus; COVID-19, coronavirus disease 2019; FHR, fetal heart rate; WBC, white blood cell; CRP, C-reactive protein.

a Abnormality of the placenta is identified as placental morphology abnormalities, placental size abnormalities, and placental position abnormalities such as placenta abruption, placenta previa, velamentous placenta, and so on.

b Abnormality of the umbilical cord include excessively long umbilical cord, excessively short umbilical cord, umbilical cord entanglement, umbilical cord knotting, umbilical cord twisting, and umbilical cord prolapse.

* *p* < 0.05.

**Table 4:** Univariate analysis of the association between perinatal factors, general clinical symptoms, and blood indices between 24 and 48 h after birth.

Variable	Abnormal WBC		Abnormal neutrophil percentage		Abnormal CRP		Abnormal composite blood indicators	
	*n*/*N* (%)	*p*-value	*n*/*N* (%)	*p*-value	*n*/*N* (%)	*p*-value	*n*/*N* (%)	*p*-value
Neonate sex		>0.99		0.797		>0.99		0.636
Male	2/55 (3.6)		20/55 (36.4)		36/55 (65.5)		17/55 (30.9)	
Female	1/41 (2.4)		13/41 (31.7)		26/41 (63.4)		10/41 (24.4)	
Mode of delivery		0.229		0.192		0.954		0.343
Vaginal delivery	1/66 (1.5)		26/66 (39.4)		42/66 (63.6)		21/66 (31.8)	
Cesarean section	2/30 (6.7)		7/30 (23.3)		20/30 (66.7)		6/30 (20.0)	
GBS positive		>0.99		0.128		0.556		0.337
Yes	0/14 (0.0)		2/14 (14.3)		8/14 (57.1)		2/14 (14.3)	
No	3/82 (3.7)		31/82 (37.8)		54/82 (65.9)		25/82 (30.5)	
Maternal diabetes mellitus		>0.99		>0.99		0.902		0.929
Yes	0/19 (0.0)		7/19 (36.8)		13/19 (68.4)		6/19 (31.6)	
No	3/77 (3.9)		26/77 (33.8)		49/77 (63.6)		21/77 (27.3)	
Abnormality of the placent^a^		>0.99		>0.99		0.318		0.720
Yes	0/10 (0.0)		3/10 (30.0)		5/10 (50.0)		2/10 (20.0)	
No	3/86 (3.5)		30/86 (34.9)		57/86 (66.3)		25/86 (29.1)	
Abnormality of the umbilical cord^b^		>0.99		0.270		0.691		0.435
Yes	1/35 (2.9)		15/35 (42.9)		24/35 (68.6)		12/35 (34.3)	
No	2/61 (3.3)		18/61 (29.5)		38/61 (62.3)		15/61 (24.6)	
Infection during pregnancy		>0.99		0.010**		>0.99		0.032*
Yes	1/29 (3.4)		16/29 (55.2)		19/29 (65.5)		13/29 (44.8)	
No	2/67 (3.0)		17/67 (25.4)		43/67 (64.2)		14/67 (20.9)	
COVID-19 infection during pregnancy		0.403		0.427		0.199		0.755
Yes	1/15 (6.7)		7/15 (46.7)		7/15 (46.7)		5/15 (33.3)	
No	2/81 (2.5)		26/81 (32.1)		55/81 (67.9)		22/81 (27.2)	
Meconium staining of amniotic fluid		0.357		0.760		>0.99		>0.99
Yes	1/13 (7.7)		5/13 (38.5)		8/13 (61.5)		3/13 (23.1)	
No	2/83 (2.4)		28/83 (33.7)		54/83 (65.1)		24/83 (28.9)	
Premature rupture of membranes >18 h		0.050*		0.405		0.582		>0.99
Yes	3/36 (8.3)		10/36 (27.8)		25/36 (69.4)		10/36 (27.8)	
No	0/60 (0.0)		23/60 (38.3)		37/60 (61.7)		17/60 (28.3)	
Application of the vacuum extractor during delivery		0.561		0.085		0.535		0.924
Yes	0/26 (0.0)		13/26 (50.0)		15/26 (57.7)		8/26 (30.8)	
No	3/70 (4.3)		20/70 (28.6)		47/70 (67.1)		19/70 (27.1)	
Intrapartum fever		>0.99		0.305		0.486		0.138
Yes	0/10 (0.0)		5/10 (50.0)		8/10 (80.0)		5/10 (50.0)	
No	3/86 (3.5)		28/86 (32.6)		54/86 (62.8)		22/86 (25.6)	
Abnormal FHR monitoring before delivery		>0.99		0.601		0.902		0.631
Yes	0/19 (0.0)		8/19 (42.1)		13/19 (68.4)		4/19 (21.1)	
No	3/77 (3.9)		25/77 (32.5)		49/77 (63.6)		23/77 (29.9)	
Maternal WBC >10 × 10^9^/L within 48 h before delivery		0.557		0.709		0.656		0.291
Yes	0/27 (0.0)		8/27 (29.6)		16/27 (59.3)		5/27 (18.5)	
No	3/69 (4.3)		25/69 (36.2)		46/69 (66.7)		22/69 (31.9)	
Neonate feeding intolerance		>0.99		0.372		0.317		0.440
Yes	0/21 (0)		5/21 (23.8)		16/21 (76.2)		4/21 (19.0)	
No	3/75 (4.0)		28/75 (37.3)		46/75 (61.3)		23/75 (30.7)	
Neonatal weight loss >3% on the first day after birth		0.565		0.226		0.747		0.295
Yes	1/23 (4.3)		5/23 (21.7)		16/23 (69.6)		4/23 (17.4)	
No	2/73 (2.7)		28/73 (38.4)		46/73 (63.0)		23/73 (31.5)	

*p*-values were calculated using Pearson's chi-squared test for comparisons between groups, and Fisher's exact test was used when any expected cell frequency was 5 or less.

GBS, group B streptococcus; COVID-19, coronavirus disease 2019; FHR, fetal heart rate; WBC, white blood cell; CRP, C-reactive protein.

a Abnormality of the placenta is identified as placental morphology abnormalities, placental size abnormalities, and placental position abnormalities such as placenta abruption, placenta previa, velamentous placenta, and so on.

b Abnormality of the umbilical cord include excessively long umbilical cord, excessively short umbilical cord, umbilical cord entanglement, umbilical cord knotting, umbilical cord twisting, and umbilical cord prolapse.

* *p* < 0.05.

** *p* < 0.01.

## 4 Discussion

This study highlights that infection indicators such as WBC count, neutrophil percentage, and CRP levels often show abnormalities in full-term infants at high risk for EOS who test negative, questioning their reliability as sole diagnostic markers. Relying solely on these indicators for antibiotic therapy in high-risk newborns may lead to overuse, emphasizing the need for more targeted approaches in neonatal care.

In diagnosing infections in children and adults, common blood infection indicators such as WBC, neutrophil percentage, and CRP are generally reliable (35–38). However, these indicators are not entirely applicable to neonatal EOS (37). Neonatologists often encounter abnormal infection indicators in well-appearing infants, creating a dilemma about whether to use antibiotics. Many studies have focused on determining cutoff values for various infection indicators to guide antibiotic use (39–43). Despite current guidelines and expert consensus suggesting that antibiotics should not be solely relied upon (21, 33), neonatologists still use these indicators to make decisions about antibiotic therapy due to the high mortality associated with EOS. Consequently, antibiotic overuse remains a significant issue in neonates. In the present study, we included 103 neonates with high-risk factors for EOS who were ultimately confirmed not to have true EOS and did not receive antibiotics during follow-up. By examining the changes in infection indicators and their relationship with perinatal factors, this study provides valuable insights into the management of neonates at risk for EOS.

In this study, 103 neonates with high-risk factors for EOS had their infection indicators tested within 24 h after birth. We found that only two neonates had normal infection indicators within this period. Relying solely on these results for antibiotic use would significantly increase the number of neonates exposed to antibiotics. The remaining 101 neonates with abnormal infection indicators at their initial assessment did not receive any antibiotics. This study reaffirms that it is not appropriate to base antibiotic treatment solely on infection indicators, as it can lead to unnecessary antibiotic exposure. Some studies have even suggested using physical examination alone to determine the need for antibiotics (22). For instance, Berardi et al. (22) compared the use of antibiotics between a group receiving laboratory evaluations plus simplified physical examination and a group receiving only physical examinations. The results showed that the group receiving only physical examinations used fewer unnecessary antibiotics and had a shorter length of stay. While in many centers, neonatologists cannot rely solely on physical examinations due to variability in the interpretation of symptoms and signs, our study at least suggests that for asymptomatic newborns, infection indicator results should not be overinterpreted.

In addition to monitoring infection indicators within the first 24 h, we continued to track these newborns until their infection indicators returned to normal. For infants with abnormal infection indicators in the first 24 h, we reexamined them within 24–48 h to rule out infection. As shown in Figure 2, while most newborns had normal white blood cell counts within 24–48 h, CRP levels exhibited different patterns. Both WBC and neutrophil percentages showed a decreasing trend from 0–24 h to 24–48 h, whereas CRP levels increased during the same period. Perrone et al. (24) examined CRP in 859 healthy term newborns during the first 48 h of life. The CRP mean values were significantly higher at 48 h. The 95th centiles for CRP at 48 h were 13.3 mg/L. This is consistent with the reference intervals established by Chiesa et al. (44), who reported that CRP levels in healthy term neonates peak during the first 48 h. In our study, the median CRP value during 24–48 h was 13.1 mg/L. This high level can be attributed to the fact that all newborns in our study had high-risk factors for infection.

Several studies have reported higher CRP levels in infants delivered vaginally compared with those delivered by cesarean section, likely reflecting increased physiological stress during labor and delivery. Vaginal delivery is associated with elevated fetal stress hormones, such as cortisol and catecholamines, which may contribute to transient increases in inflammatory markers including CRP and white blood cell indices (24, 44–49). In line with these observations, our data showed that although WBC counts and neutrophil percentages generally decreased between 24 and 48 h after birth, CRP levels often rose during the same period. Notably, 28 infants had normal CRP levels within the first 24 h but developed elevated CRP values at 24–48 h, despite the absence of clinical infection. Therefore, isolated CRP elevation, particularly in the context of improving WBC indices, should be interpreted with caution in clinical decision-making.

Despite their limited specificity, inflammatory markers such as CRP and white blood cell indices are still widely used by neonatologists to guide decisions regarding antibiotic initiation, given the potentially high mortality associated with early-onset sepsis. However, De Rose et al. (50) reported that CRP levels at admission were similar in neonates with positive and negative blood cultures and demonstrated that, in clinically stable infants, antibiotic therapy may be safely discontinued when blood cultures remain negative after 36–48 h. Together, these findings underscore the challenge of interpreting isolated or delayed CRP elevation in high-risk but clinically well-term infants.

To further explore potential non-infectious contributors to this pattern, we examined the role of delivery mode on inflammatory markers. Perrone et al.'s (24) study also noted that newborns delivered vaginally had elevated CRP levels, which aligns with our findings regarding WBC (*p* = 0.052, Figure 3). This is consistent with previous reports indicating that the physiologic stress of labor and vaginal delivery may contribute to a more pronounced acute-phase response in the newborn, even in the absence of infection. Furthermore, a similar trend was observed between vacuum-assisted delivery and elevated neutrophil percentages (*p* = 0.074). It should be noted that these associations are observational in nature and, as indicated by their *p*-values, are suggestive rather than statistically conclusive. Although these specific associations did not reach conventional statistical significance in our cohort, they collectively suggest that the mode of delivery, including operative vaginal delivery, may act as a non-infectious stressor influencing early neonatal hematological indices. These findings suggest that vacuum-assisted delivery may be associated with transient elevations in inflammatory markers reflecting physiological stress, rather than indicating true neonatal infection. Thus, these findings are best interpreted as generating hypotheses for future investigation. Such physiological elevations underscore the challenge of interpreting isolated CRP increases in the early neonatal period, particularly in high-risk but clinically well infants. Previous studies have shown that up to 20% of healthy newborns may exhibit a physiological rise in CRP above 10 mg/L, and even up to 20 mg/L, during the first days after birth. Benitz et al. (51) and Hengst (52) reported that such transient CRP elevations may occur in the absence of infection and should be interpreted in conjunction with the infant's overall clinical condition rather than in isolation. This physiological pattern further supports cautious interpretation of elevated CRP values in clinically well neonates.

However, it should be noted that neonatal weight loss >3% on the first day after birth is not an established risk factor or indicator of neonatal infection. Beyond the mode of delivery, other perinatal factors were also linked to abnormalities in infection markers. For instance, premature rupture of membranes for >18 h was identified as a risk factor for abnormal WBC levels within the first 24 h. Similarly, feeding intolerance or weight loss exceeding 3% in newborns emerged as a significant factor associated with elevated CRP. While these perinatal conditions do not definitively rule out infection—and infants exhibiting such factors alongside abnormal markers warrant close observation—their association with altered hematological indices serves as an important clinical reminder. These findings suggest that the possibility of a non-infectious etiology should be actively considered in such scenarios, which could help mitigate unnecessary antibiotic use.

According to the NICE guideline (20, 21), symptoms such as breathing difficulties, tachycardia, and seizures are considered significant indicators or red flags for sepsis. In line with this, our protocol involved active monitoring for a comprehensive set of EOS symptoms across all major organ systems (respiratory, cardiovascular, neurological, gastrointestinal, metabolic, and thermoregulatory). In our study, we also examined these symptoms to explore their relationship with EOS. However, aside from digestive symptoms (such as feeding intolerance or weight loss exceeding 3%), these other symptoms were not observed in any of the 103 newborns, resulting in an incidence rate of 0%. This is consistent with our cohort's defining characteristic: All infants were clinically well-appearing and managed in the rooming-in ward. Per our clinical protocol, any neonate exhibiting systemic signs of illness (e.g., apnea, tachycardia, and respiratory distress) would have been transferred to the NICU for evaluation and thus excluded from this analysis. This absence of symptoms is likely because infants presenting with such signs would have been admitted to the NICU for further evaluation of potential systemic diseases. In the NICU, these infants would have undergone more rigorous monitoring and additional assessments to determine the presence of EOS.

In addition, jaundice within the first 24 h after birth is also considered an indicator or high-risk factor for EOS. We included jaundice in our evaluation of the 103 neonates. For the diagnosis of jaundice, our data included the expert consensus of Chinese local experts (53). The standard for 24 h jaundice is based on the hourly bilirubin curve, and any value above this standard is defined as 24 h jaundice. However, this factor did not show any significant association with the abnormalities in the three infection indicators. The latest guidelines from the American Academy of Pediatrics (54) have updated the criteria for jaundice, and what was previously considered abnormal is no longer classified as jaundice. According to these updated guidelines, only two newborns met the criteria for 24 h jaundice. Due to the small sample size and its minimal impact, we did not include this factor in our study.

Widely accepted high-risk factors for EOS include GBS colonization, chorioamnionitis, and preterm birth (3–6). In this study, all mothers who tested positive for GBS received adequate antibiotics before delivery. Infants whose mothers had not received sufficient antibiotics were closely monitored in the NICU, rather than in rooming-in. Infants diagnosed with chorioamnionitis were also monitored in the NICU. For preterm birth, only partially preterm infants with >36 gestation weeks were admitted to rooming-in. If infants are born at <36 weeks, they would admit in NICU for further monitoring in our center. Consequently, we explored the application of antibiotics in all late preterm infants due to the differing care practices between the NICU and rooming-in settings. Therefore, preterm births were not included in the present study.

Some guidelines recommend managing multiple positive risk factors for EOS (33, 55). In this study, we analyzed maternal GBS positivity, intrapartum fever, and premature rupture of membranes for >18 h. However, there were no cases with maternal GBS positivity combined with both premature rupture of membranes for >18 h and intrapartum fever. We identified two cases with any two of these risk factors. According to the local guideline (33), the presence of two positive risk factors suggests transferring neonates to a specialized department and considering antibiotic use. Although antibiotics were not administered in these two cases, no definitive conclusions can be drawn from such a small sample. This finding warrants further investigation with a larger sample size in future studies.

Several limitations must be acknowledged when interpreting our findings: Firstly, the relatively small sample size limited statistical power, particularly in multivariate analyses. Observed correlations should therefore be interpreted as hypothesis-generating and require validation in larger cohorts. Secondly, the single-center retrospective design introduces a risk of selection bias, which may compromise the external validity of the statistical inferences. Thirdly, although efforts were made to adjust for known confounders, there remains a possibility that unmeasured biases and confounders were not sufficiently addressed in the statistical models. Fourthly, our study specifically focused on neonates at high risk for EOS who were ultimately confirmed to be uninfected and managed in the rooming-in ward. While this design allowed us to characterize infection indicators in the absence of antibiotic confounding, it also means that our cohort comprised infants who were, in retrospect, truly low risk for developing EOS. This may limit the direct generalizability of our findings to all high-risk infants, particularly those who present with more equivocal clinical signs. Nevertheless, this population remains highly relevant to clinical practice. In many settings, including numerous NICUs in developing countries, the decision to initiate antibiotics in asymptomatic or minimally symptomatic high-risk infants is still heavily influenced by abnormal laboratory indicators. Our findings directly address this common clinical dilemma by demonstrating the high frequency of such abnormalities in infants who do not have infection, thereby underscoring the potential for antibiotic overuse in this specific group.

Our findings directly inform antimicrobial stewardship efforts by demonstrating that abnormal WBC, neutrophil%, and CRP values are common in high-risk but uninfected term infants. This underscores the risk of antibiotic overuse when these markers are interpreted in isolation, supporting a more conservative, clinically integrated approach to antibiotic initiation in asymptomatic neonates. In conclusion, this study reaffirms that relying solely on blood infection indicators is insufficient for accurately diagnosing neonatal EOS. In newborns identified as high-risk for EOS but ultimately confirmed not to have the condition, only a few exhibited normal WBC count, neutrophil percentage, and CRP levels within 24 h after birth. While WBC levels may return to the normal range within 24–48 h, CRP levels often remain elevated. Additionally, certain perinatal factors, such as feeding intolerance, have been identified as risk factors for abnormalities in blood infection indicators. Further exploration is needed to determine the significance of multiple positive risk factors for EOS as an indication for antibiotic administration.

## Data Availability

The raw data supporting the conclusions of this article will be made available by the authors, without undue reservation.

## References

[1] Giannoni E Agyeman PKA Stocker M Posfay-Barbe KM Heininger U Spycher BD Neonatal sepsis of early onset, and hospital-acquired and community-acquired late onset: a prospective population-based cohort study. J Pediatr. (2018) 201:106–14.e4. 10.1016/j.jpeds.2018.05.048 30054165

[2] Achten NB Juliana AE Lissone NP Sinnige JC Holband N Zonneveld R Epidemiology and mortality of early-onset neonatal sepsis in Suriname: a 2-year surveillance study. J Pediatric Infect Dis Soc. (2021) 10(4):514–6. 10.1093/jpids/piaa130 33231629

[3] Polin RA. Management of neonates with suspected or proven early-onset bacterial sepsis. Pediatrics. (2012) 129(5):1006–15. 10.1542/peds.2012-0541 22547779

[4] Shane AL Sánchez PJ Stoll BJ. Neonatal sepsis. Lancet. (2017) 390(10104):1770–80. 10.1016/S0140-6736(17)31002-4 28434651

[5] Puopolo KM Benitz WE Zaoutis TE. Management of neonates born at ≥35 0/7 weeks’ gestation with suspected or proven early-onset bacterial sepsis. Pediatrics. (2018) 142(6):e20182894. 10.1542/peds.2018-2894 30455342

[6] Kuzniewicz MW Puopolo KM Fischer A Walsh EM Li S Newman TB A quantitative, risk-based approach to the management of neonatal early-onset sepsis. JAMA Pediatr. (2017) 171(4):365–71. 10.1001/jamapediatrics.2016.4678 28241253

[7] Raymond SL Rincon JC Wynn JL Moldawer LL Larson SD. Impact of early-life exposures to infections, antibiotics, and vaccines on perinatal and long-term health and disease. Front Immunol. (2017) 8:729. 10.3389/fimmu.2017.00729 28690615 PMC5481313

[8] Reyman M van Houten MA Watson RL Chu M Arp K de Waal WJ Effects of early-life antibiotics on the developing infant gut microbiome and resistome: a randomized trial. Nat Commun. (2022) 13(1):893. 10.1038/s41467-022-28525-z 35173154 PMC8850541

[9] McDonnell L Gilkes A Ashworth M Rowland V Harries TH Armstrong D Association between antibiotics and gut microbiome dysbiosis in children: systematic review and meta-analysis. Gut Microbes. (2021) 13(1):1–18. 10.1080/19490976.2020.1870402 33651651 PMC7928022

[10] Stocker M Klingenberg C Navér L Nordberg V Berardi A El Helou S Less is more: antibiotics at the beginning of life. Nat Commun. (2023) 14(1):2423. 10.1038/s41467-023-38156-7 37105958 PMC10134707

[11] Ng SC Bernstein CN Vatn MH Lakatos PL Loftus EV Jr Tysk C Geographical variability and environmental risk factors in inflammatory bowel disease. Gut. (2013) 62(4):630–49. 10.1136/gutjnl-2012-303661 23335431

[12] Aversa Z Atkinson EJ Schafer MJ Theiler RN Rocca WA Blaser MJ Association of infant antibiotic exposure with childhood health outcomes. Mayo Clin Proc. (2021) 96(1):66–77. 10.1016/j.mayocp.2020.07.019 33208243 PMC7796951

[13] Renz H Skevaki C. Early life microbial exposures and allergy risks: opportunities for prevention. Nat Rev Immunol. (2021) 21(3):177–91. 10.1038/s41577-020-00420-y 32918062

[14] World Health Organization. Global action plan on antimicrobial resistance: options for establishing a global development and stewardship framework to support the development, control, distribution and appropriate use of new antimicrobial medicines, diagnostic tools, vaccines and other interventions. (2016). Available online at: https://apps.who.int/iris/bitstream/handle/10665/252682/A69_24Add1en.pdf?sequence=1&isAllowed=y (Accessed July 2024).

[15] Iroh Tam PY Bendel CM. Diagnostics for neonatal sepsis: current approaches and future directions. Pediatr Res. (2017) 82(4):574–83. 10.1038/pr.2017.134 28574980

[16] Ng PC. Diagnostic markers of infection in neonates. Arch Dis Child Fetal Neonatal Ed. (2004) 89(3):F229–35. 10.1136/adc.2002.023838 15102726 PMC1721679

[17] Connell TG Rele M Cowley D Buttery JP Curtis N. How reliable is a negative blood culture result? Volume of blood submitted for culture in routine practice in a children’s hospital. Pediatrics. (2007) 119(5):891–6. 10.1542/peds.2006-0440 17473088

[18] van Herk W Stocker M van Rossum AMC. Recognising early onset neonatal sepsis: an essential step in appropriate antimicrobial use. J Infect. (2016) 72:S77–82. 10.1016/j.jinf.2016.04.026 27222092

[19] Vergnano S Menson E Kennea N Embleton N Russell AB Watts T Neonatal infections in England: the NeonIN surveillance network. Arch Dis Child Fetal Neonatal Ed. (2011) 96(1):F9–14. 10.1136/adc.2009.178798 20876594

[20] Paul SP Khattak H Kini PK Heaton PA Goel N. NICE guideline review: neonatal infection: antibiotics for prevention and treatment (NG195). Arch Dis Childhood Educ Pract Ed. (2022) 107(4):292–7. 10.1136/archdischild-2021-322349 34772670

[21] National Institute for Health and Care Excellence guideline. Neonatal infection: antibiotics for prevention and treatment (2021). Available online at: https://www.nice.org.uk/guidance/ng195 (Accessed July 2024).

[22] Berardi A Fornaciari S Rossi C Patianna V Bacchi Reggiani ML Ferrari F Safety of physical examination alone for managing well-appearing neonates ≥35 weeks’ gestation at risk for early-onset sepsis. J Matern Fetal Neonatal Med. (2015) 28(10):1123–7. 10.3109/14767058.2014.946499 25034325

[23] Escobar GJ Li DK Armstrong MA Gardner MN Folck BF Verdi JE Neonatal sepsis workups in infants ≥2000 grams at birth: a population-based study. Pediatrics. (2000) 106(2 Pt 1):256–63. 10.1542/peds.106.2.256 10920148

[24] Perrone S Lotti F Longini M Rossetti A Bindi I Bazzini F C reactive protein in healthy term newborns during the first 48 h of life. Arch Dis Child Fetal Neonatal Ed. (2018) 103(2):F163–6. 10.1136/archdischild-2016-312506 28667188

[25] Chaudhuri PK Ghosh A Sinha V Singh BK Singh M Lugova H The role of C-reactive protein estimation in determining the duration of antibiotic therapy in neonatal sepsis. Cureus. (2022) 14(10):e30211. 10.7759/cureus.30211 36246087 PMC9554835

[26] Yektaei-Karin E Moshfegh A Lundahl J Berggren V Hansson LO Marchini G. The stress of birth enhances *in vitro* spontaneous and IL-8-induced neutrophil chemotaxis in the human newborn. Pediatr Allergy Immunol. (2007) 18(8):643–51. 10.1111/j.1399-3038.2007.00578.x 18078418

[27] Bitar L Stonestreet BS Chalak LF. Key inflammatory biomarkers in perinatal asphyxia: a comprehensive review. Clin Perinatol. (2024) 51(3):617–28. 10.1016/j.clp.2024.04.004 39095100

[28] Cakir U Tugcu AU Tayman C Yildiz D. Evaluation of the effectiveness of systemic inflammatory indices in the diagnosis of respiratory distress syndrome in preterm with gestational age of ≤32 weeks. Am J Perinatol. (2024) 41(S 01):e1546–52. 10.1055/a-2051-8544 36898408

[29] Evidence-Based Medicine Group, Neonatologist Society, Chinese Medical Doctor Association. Clinical guidelines for the diagnosis and treatment of feeding intolerance in preterm infants (2020). Chin J Contemp Pediatr. (2020) 22(10):1047–55. 10.7499/j.issn.1008-8830.2008132 PMC7568993 33059799

[30] The Subspecialty Group of Neonatology, the Society of Pediatric, Chinese Medical Association Professional Committee of Infectious Diseases, Neonatology Society, Chinese Medical Doctor Association. Expert consensus on the diagnosis and management of neonatal sepsis (version 2019). Chin J Pediatr. (2019) 57(4):252–7. 10.3760/cma.j.issn.0578-1310.2019.04.005 30934196

[31] Celik IH Hanna M Canpolat FE Mohan P. Diagnosis of neonatal sepsis: the past, present and future. Pediatr Res. (2022) 91(2):337–50. 10.1038/s41390-021-01696-z 34728808 PMC8818018

[32] Manroe BL Weinberg AG Rosenfeld CR Browne R. The neonatal blood count in health and disease. I. Reference values for neutrophilic cells. J Pediatr. (1979) 95(1):89–98. 10.1016/S0022-3476(79)80096-7 480023

[33] Neonatal Health Care Committee of Chinese Maternal and Child Health Association, Neonatology Society of Chinese Medical Doctor Association. Expert consensus on clinical management of newborns at high risk of early-onset infection at rooming-in ward. Chin J Perinat Med. (2021) 24(8):567–75. 10.3760/cma.j.cn113903-20210310-00202

[34] Stocker M Giannoni E. Game changer or gimmick: inflammatory markers to guide antibiotic treatment decisions in neonatal early-onset sepsis. Clin Microbiol Infect. (2024) 30(1):22–7. 10.1016/j.cmi.2023.02.021 36871829

[35] Yang X Zeng J Yu X Wang Z Wang D Zhou Q PCT, IL-6, and IL-10 facilitate early diagnosis and pathogen classifications in bloodstream infection. Ann Clin Microbiol Antimicrob. (2023) 22(1):103. 10.1186/s12941-023-00653-4 37986183 PMC10662675

[36] Carter MJ Carrol ED Ranjit S Mozun R Kissoon N Watson RS Susceptibility to childhood sepsis, contemporary management, and future directions. Lancet Child Adolescent Health. (2024) 8(9):682–94. 10.1016/S2352-4642(24)00141-X 39142742

[37] Strunk T Molloy EJ Mishra A Bhutta ZA. Neonatal bacterial sepsis. Lancet. (2024) 404(10449):277–93. 10.1016/S0140-6736(24)00495-1 38944044

[38] Seymour CW Rosengart MR. Septic shock: advances in diagnosis and treatment. JAMA. (2015) 314(7):708–17. 10.1001/jama.2015.7885 26284722 PMC4646706

[39] van Leeuwen LM Fourie E van den Brink G Bekker V van Houten MA. Diagnostic value of maternal, cord blood and neonatal biomarkers for early-onset sepsis: a systematic review and meta-analysis. Clin Microbiol Infect. (2024) 30(7):850–7. 10.1016/j.cmi.2024.03.005 38467246

[40] Stocker M van Herk W El Helou S Dutta S Schuerman F van den Tooren-de Groot RK C-reactive protein, procalcitonin, and white blood count to rule out neonatal early-onset sepsis within 36 hours: a secondary analysis of the neonatal procalcitonin intervention study. Clin Infect Dis. (2021) 73(2):e383–90. 10.1093/cid/ciaa876 32881994

[41] Stocker M van Herk W El Helou S Dutta S Fontana MS Schuerman F Procalcitonin-guided decision making for duration of antibiotic therapy in neonates with suspected early-onset sepsis: a multicentre, randomised controlled trial (NeoPIns). Lancet. (2017) 390(10097):871–81. 10.1016/S0140-6736(17)31444-7 28711318

[42] Kung E Unterasinger L Waldhor T Berger A Wisgrill L. Cut-off values of serum interleukin-6 for culture-confirmed sepsis in neonates. Pediatr Res. (2023) 93(7):1969–74. 10.1038/s41390-022-02329-9 36216867 PMC10313515

[43] Hornik CP Benjamin DK Becker KC Benjamin DK Jr Li J Clark RH Use of the complete blood cell count in early-onset neonatal sepsis. Pediatr Infect Dis J. (2012) 31(8):799–802. 10.1097/INF.0b013e318256905c 22531231 PMC3399972

[44] Chiesa C Natale F Pascone R Osborn JF Pacifico L Bonci E C reactive protein and procalcitonin: reference intervals for preterm and term newborns during the early neonatal period. Clin Chim Acta. (2011) 412(11-12):1053–9. 10.1016/j.cca.2011.02.020 21338596

[45] Tiozzo C Mukhopadhyay S. Noninfectious influencers of early-onset sepsis biomarkers. Pediatr Res. (2022) 91(2):425–31. 10.1038/s41390-021-01861-4 34802035 PMC8818022

[46] Bellieni CV Liuzzo LP Giomi S Tei M Stazzoni G Bertrando S C-reactive protein: a marker of neonatal stress? J Matern Fetal Neonatal Med. (2014) 27(6):612–5. 10.3109/14767058.2013.823937 23859542

[47] Malamitsi-Puchner A Protonotariou E Boutsikou T Makrakis E Sarandakou A Creatsas G. The influence of the mode of delivery on circulating cytokine concentrations in the perinatal period. Early Hum Dev. (2005) 81(4):387–92. 10.1016/j.earlhumdev.2004.10.017 15814224

[48] Kääpä P Koistinen E. Maternal and neonatal C-reactive protein after interventions during delivery. Acta Obstet Gynecol Scand. (1993) 72(7):543–6. 10.3109/00016349309058160 8213101

[49] Ishibashi M Takemura Y Ishida H Watanabe K Kawai T. C-reactive protein kinetics in newborns: application of a high-sensitivity analytic method in its determination. Clin Chem. (2002) 48(7):1103–6. 10.1093/clinchem/48.7.1103 12089183

[50] De Rose DU Perri A Auriti C Gallini F Maggio L Fiori B Time to positivity of blood cultures could inform decisions on antibiotics administration in neonatal early-onset sepsis. Antibiotics. (2021) 10(2):123. 10.3390/antibiotics10020123 33525647 PMC7910918

[51] Benitz WE Han MY Madan A Ramachandra P. Serial serum C-reactive protein levels in the diagnosis of neonatal infection. Pediatrics. (1998) 102(4):E41. 10.1542/peds.102.4.e41 9755278

[52] Hengst JM. The role of C-reactive protein in the evaluation and management of infants with suspected sepsis. Adv Neonatal Care. (2003) 3(1):3–13. 10.1053/adnc.2003.50010 12882177

[53] The Subspecialty Group of Neonatology of the Society of Pediatrics of Chinese Medical Association, Chinese Journal of Pediatrics Editorial Board. Expert consensus on diagnosis and treatment of neonatal hyperbilirubinemia. Chin J Pediatr. (2014) 52(10):745–8. 25537539

[54] Kemper AR Newman TB Slaughter JL Maisels MJ Watchko JF Downs SM Clinical practice guideline revision: management of hyperbilirubinemia in the newborn infant 35 or more weeks of gestation. Pediatrics. (2022) 150(3):e2022058859. 10.1542/peds.2022-058859 35927462

[55] Verani JR McGee L Schrag SJ. Prevention of perinatal group B streptococcal disease–revised guidelines from CDC, 2010. MMWR Recommend Rep. (2010) 59(Rr-10):1–36. 21088663

